# The Relationship Between Heart Rate Variability and Electroencephalography Functional Connectivity Variability Is Associated With Cognitive Flexibility

**DOI:** 10.3389/fnhum.2019.00064

**Published:** 2019-02-25

**Authors:** Guzmán Alba, Jaime Vila, Beatriz Rey, Pedro Montoya, Miguel Ángel Muñoz

**Affiliations:** ^1^Brain, Mind and Behavior Research Center, University of Granada, Granada, Spain; ^2^Departamento de Ingeniería Gráfica, Universitat Politècnica de València, Valencia, Spain; ^3^Research Institute of Health Sciences, University of Balearic Islands, Palma, Spain

**Keywords:** HRV, EEG, resting-state, sample entropy, variability of functional connectivity, cognitive flexibility

## Abstract

The neurovisceral integration model proposes a neuronal network that is related to heart rate activity and cognitive performance. The aim of this study was to determine whether heart rate variability (HRV) and variability in electroencephalographic (EEG) functional connectivity in the resting state are related to cognitive flexibility. Thirty-eight right-handed students completed the CAMBIOS test, and their heart and EEG activity was recorded during 6 min in the resting state with their eyes open. We calculated correlations, partial correlations and multiple linear regressions among HRV indices, functional brain connectivity variability and CAMBIOS scores. Furthermore, the sample was divided into groups according to CAMBIOS performance, and one-way ANOVA was applied to evaluate group differences. Our results show direct and inverse correlations among cognitive flexibility, connectivity (positive and negative task networks) and heartbeat variability. Partial correlations and multiple linear regressions suggest that the relation between HRV and CAMBIOS performance is mediated by neuronal oscillations. ANOVA confirms that HRV and variability in functional brain connectivity is related to cognitive performance. In conclusion, the levels of brain signal variability might predict cognitive flexibility in a cognitive task, while HRV might predict cognitive flexibility only when it is mediated by neuronal oscillations.

## Introduction

Cognitive flexibility refers to the ability to flexibly adapt processing to changing environmental information, to guide thought and behavior and to allow directed action toward a goal (Cañas et al., 2003; Geurts et al., 2009; Dennis and Vander Wal, 2010; Ionescu, 2012). Flexibility depends on strong executive control, particularly in terms of efficient shifting of attentional and cognitive resources to the processing of new information while inhibiting previous irrelevant information (Miyake et al., 2000). Research has shown a direct link between cognitive flexibility and the cardiovascular system through the autonomic vagal tone. A simple way of measuring this relationship is to examine heart rate variability (HRV), a non-invasive measurement of the interactions between the autonomic nervous system (ANS) and central nervous system (CNS) based on the study of oscillations of the interval between heartbeats (Malik et al., 1996; Pumprla et al., 2002). The neurovisceral integration model (Thayer and Lane, 2009) proposes a neuronal network that relates heart rate activity and cognitive performance. This model assumes that the CNS and ANS are reciprocally interconnected such that information flows bidirectionally (Smith et al., 2017). Very compelling evidence indicates that prefrontal cortex activity is involved in the modulation of vagal efferent outflow to the heart, and HRV is an indicator of cardiac activity associated with cognitive flexibility in tasks involving attention, working memory and inhibitory control (Thayer et al., 2004; Saus et al., 2006; Hansen et al., 2009). The high-frequency component of HRV appears to be positively correlated with perseverative errors in the Wisconsin Card Sorting Test, as well as inhibition errors in the Color-Word Interference Test (Hovland et al., 2012). In a series of studies in which individuals were *a priori* stratified according to their resting-state levels of HRV, individuals with high HRV performed better on tasks involving executive function than those with low HRV (Hansen et al., 2009). Such findings indicate that individual differences in HRV are a useful predictor of cognitive flexibility (Gillie and Thayer, 2014).

Lately, brain signal variability has emerged as a valuable tool for investigating individual differences in cognitive performance (Yang et al., 2013; McDonough and Nashiro, 2014). The capacity of resting-state functional connectivity variability to predict cognitive performance with different methods and tasks has been explored (Mennes et al., 2010; Takeuchi et al., 2011; Mackey et al., 2013; Martínez et al., 2013; Thompson et al., 2016). These studies seem to indicate that executive functions are correlated with brain activity in the resting state (Fox et al., 2005, 2006; Seeley et al., 2007). Mennes et al. (2010) found a task-positive network that included frontal-cingulate-parietal areas during an Eriksen Flanker task. Fox et al. (2006) identified two brain networks in the resting state. One network consists of regions that are routinely positively correlated with cognitive task performance, and the other includes regions that are routinely negatively correlated. The presence of significant positive/negative correlations between a cerebral region and a task across participants suggests that at least some part of the cerebral response induced by a particular task is intrinsically represented in the brain (Mennes et al., 2010). Similarly, when variability is disrupted, the brain has little capacity to adapt to environmental conditions, resulting in neuropathological diseases such as epilepsy and attention-deficit/hyperactivity disorder (Mizuno et al., 2010; Catarino et al., 2011; Vakorin et al., 2011; Ramon and Holmes, 2013; Barttfeld et al., 2014; Alba et al., 2016; Chen et al., 2017).

Recently, relations between HRV and the endogenous dynamic of brain regions involved in autonomic control and emotional regulation during the resting state have been explored. These studies showed that high- and low-frequency components of HRV are strongly coupled with functional connectivity (Chang et al., 2013b; Jennings et al., 2016; Sakaki et al., 2016). However, these studies have not addressed the relation between the variability of functional connectivity and HRV or whether both factors might predict the outcomes of cognitive tasks. Palva et al. (2013) correlated the variability of functional connectivity using magneto-encephalography (MEG) and HRV during a stimulus detection task and during the resting-state period. Strong correlations were found between neuronal oscillations and task performance during the task and during the resting-state period. These results suggest that the variability of functional connectivity in the resting state is not specific to the task but is related to the performance of cognitive tasks. Moreover, this study found that HRV in both task and rest conditions predicted task performance.

In the present study, we investigated the association between electroencephalography (EEG) functional connectivity variability and HRV in the resting state and subsequent performance on a cognitive test. Normally, any EEG measure is estimated during the resting state by averaging its values in a certain number of EEG segments. This procedure assumes that resting-state EEG remains static during the recording period. Even in this case, recent evidence (see, e.g., Kitzbichler et al., 2009; Botcharova et al., 2014) clearly suggests that brain synchronization as assessed from neurophysiological signals is not constant, but during this time, it presents significant variability, which is disrupted in neuropathological conditions (Ramon and Holmes, 2013; Alba et al., 2016). Moreover, some studies suggest that the variability of functional connectivity in the resting state could predict cognitive performance (Liu et al., 2018). This association between variability and variations in mental state seems to oscillate at different frequency bands, such as theta or alpha (Chang et al., 2013a). Research on the variability of functional connectivity can unveil flexibility in the functional coordination between different neuronal systems and may improve our understanding of behavioral shifts and adaptive processes (Allen et al., 2014). In other words, how brain connectivity dynamics in the resting state might predict the outcome of cognitive tasks is of interest.

The aim of this study was to determine whether performance in a switch task is correlated with the interindividual variability of functional connectivity in the resting state and with HRV. Thus, we expect that better performance on the CAMBIOS switch test will be related to high variability of functional connectivity in the resting state. Furthermore, we expect that HRV and cognitive performance will be correlated. Because the literature regarding the relationship between HRV and cognitive performance shows contradictory results, we do not predict correlations or anticorrelations between these variables. Also, we hypothesize that the resting-state variability of functional connectivity (RSVFC) and HRV are correlated because the CNS and ANS are reciprocally interconnected. Moreover, to test whether fluctuations driven by the ANS play a role in the correlation between cerebral functional connectivity in the resting state and cognitive performance, we calculated the effect of HRV on correlations between performance in terms of CAMBIOS test scores and RSVFC using partial correlations. Following the previous results, we hypothesize that participants with better scores on the CAMBIOS test would show higher RSVFC. Based on previous studies, we have no hypothesis regarding the HRV level (higher or lower) of participants with higher scores on the CAMBIOS test.

## Materials and Methods

### Participants

Thirty-eight right-handed students (22 females and 16 males) participated in this study. All students attended the University of Granada (average age = 20.82 ± 9.5) and received extra credit in return for their participation. Participant exclusion criteria included cardiovascular problems, ongoing illicit substance use, mental health problems, or current medical or psychological treatment. We excluded four participants (2 females and 2 males) from the study due to technical issues with their recordings. Participants were recruited via information provided in university classrooms. All participants signed informed consent forms to participate in the study, which was approved by the ethics committee of University of Granada and carried out according to the recommendations of the Declaration of Helsinki.

### Procedure

Data were compiled in an individual session that lasted approximately 45 min. Upon their arrival at the experimental session, participants received a brief explanation of the study before signing their informed consent, followed by a short interview to verify compliance with the inclusion criteria. Then, participants performed the CAMBIOS test, and they were moved to the recording room (quiet and dimly illuminated) and sat in a comfortable seat. Next, EEG and electrocardiography (EKG) electrodes were applied, and the experimental session started. Recordings were performed during a 3 min adaptation period and a 6 min resting-state period with eyes open. All participants were instructed to fix their eyes on a fixation cross to reduce ocular artifacts. Finally, a 3 min period to allow participants to return to the basal level was included. The instructions for the experimental conditions and fixation cross were presented with E-Prime 2.0 software (Psychology Software Tools) and a Canon LV-53 projector.

### Cognitive Test

The CAMBIOS test (Seisdedos, 2004) evaluates cognitive flexibility. Cognitive flexibility can be described as open, organized, systematic behavior and the ability to quickly respond to classification stimuli. During the CAMBIOS test, participants determine whether there has been a change between three polygonal figures according to easily learned symbology. Participants had 7 min to complete the test. Two indices were used in analysis, errors (number of errors/total number of items) and hits (number of hits/total number of items).

### Physiological Data Acquisition and Preprocessing

Physiological signals from EKG and EEG were continuously acquired using Ag/AgCl electrodes and two electro-oculogram channels with a 32-ch A.N.T. EEG System (Enschede, Netherlands). The sampling rate was accomplished at a frequency of 1024 Hz. Electrodes were mounted according to the 10/20 montage system, and a bilateral electro-oculogram was recorded from horizontal sites to monitor blinking and was referenced to ear lobes. All EEG channels were offline-referenced to the average of the electrodes, and impedance was maintained at <10 kOhm. Two-lead EKG was recorded using 8 mm Ag/AgCl surface electrodes placed over the wrists and ankles of participants and filtered with cut-offs above 5 Hz and below 35 Hz.

EEG preprocessing analysis was performed with the EEGLAB toolbox for MATLAB (Delorme and Makeig, 2004). The recordings were resampled to 512 Hz and filtered with a 0.01–40 Hz bandpass filter. Eye movement and blink artifacts were corrected using independent component analysis. EEG waveforms were segmented in epochs of 1 s duration (obtained a total of 360 epochs) for analyses, and artifact rejection methods consisted of exclusion of epochs with large amplitudes (over ± 100 μV). After artifact rejection was applied, the largest recording had 300 epochs, and the shortest recording had 180 epochs. The mean number of epochs rejected was 39.21, and the standard deviation was 34.20. To equalize the number of epochs between participants, we selected the first 180 epochs after rejection in all recordings. Because the literature indicates that cognitive performance is related to some frequency bands (Carrillo-de-la-Peña and García-Larrea, 2007; Cooper et al., 2015; Sadaghiani and Kleinschmidt, 2016), we explored the variability of EEG functional connectivity in the frequency domain at delta (1–4 Hz), theta (4–8 Hz), alpha (8–13 Hz), and beta (13–30 Hz).

HR was estimated from the R–R intervals from EKG filtering using the ECGLAB toolbox for MATLAB (de Carvalho et al., 2002). Cardiac autonomic activity was obtained from power spectral analysis of the R–R interval, named HRV by KARDIA software (Perakakis et al., 2010). We calculated the following HRV measures: high frequency (HF), low frequency (LF), very low frequency (VLF), RMSSD, and standard deviation of NN intervals (SDNN).

### Variability in Resting-State Functional Connectivity

EEG variability functional connectivity analysis was conducted with a MATLAB self-programmed script. The procedure used to study RSVFC was as follows. First, coherence was calculated as the functional connectivity index (FC) in each frequency band (delta, theta, alpha and beta). This index measures the linear correlation between two EEG signals, x(t) and y(t), as a function of the frequency, f. Thus, coherence (C) is the ratio of the cross-power spectral density, *Sxy(f)*, between both signals and their individual power spectral densities, *Sxx(f)* and *Syy(f)*:

(1) $$C_{xy}(f)=\frac{S_{xy}(f)}{\sqrt{S_{xx}(f)S_{yy}(f)}}$$

To reject spurious correlations between cortical sources, the imaginary part of coherence (IC) was extracted. The IC was calculated in each EEG epoch between pairs of electrodes, yielding 435 links.

Finally, the variability of IC was calculated across 180 epochs in each frequency using the sample entropy algorithm (Richman and Moorman, 2000). The sample entropy of FC (SampEn-FC) allows us to obtain the variability of connectivity over time and the interdependencies between pairs of nodes (electrodes) in brain networks. SampEn is the negative logarithm of the conditional probability that two sequences will remain similar at the next point (2) where self-matches are not included in calculating the probability.

(2) $$\mathrm{SampEn}(m,\;r,\;N)=-\ln\left[\frac{U^{m+1}(r)}{U^{m}(r)}\right]$$

Where *m* is the embedded dimension, *r* is the tolerance value and *N* is the time series data. In the current work, *m* = 2 and *r* = 0.15^∗^Standard Deviation.

### Statistical Analysis

SampEn-FC, HRV, and CAMBIOS scores were initially checked for a normal distribution by using the Shapiro–Wilk test. Because these measurements significantly deviated from a normal distribution, correlations were assessed by using Spearman correlations.

To study how performance on a switching task is correlated with interindividual RSVFC and with HRV, we analyzed Spearman’s correlations between CAMBIOS (Error and Hit scores) and HRV indices (HF, LF, VLF, RMSSD, and SDNN) and between CAMBIOS and SampEn-FC links in each frequency (delta, theta, alpha, and beta). Previous studies have demonstrated that the brain cognitive performance can be functionally divided into two networks: task-positive and task-negative networks (Jia et al., 2014). We built positive and negative networks in each frequency band. The positive network consisted of links with positive correlations between SampEn-FC and CAMBIOS scores. The negative network consisted of links with negative correlations between SampEn-FC and CAMBIOS scores. Finally, SampEn-FC means were obtained from the whole brain in each positive and negative network of all frequency bands, and partial correlations were calculated to measure the degree of association between the SampEn-FC means of the EEG networks, HRV and CAMBIOS scores.

To obtain further insight into the pattern of associations, multiple regression analyses were conducted, with EEG networks (in each frequency band) and HRV indices as predictors and CAMBIOS scores as the dependent variable. Eight separate regression analyses for each task network (positive and negative) and frequency band (delta, theta, alpha, and beta) were computed. A “stepwise” procedure was applied for the entry and removal of predictors. In this method, the predictor which explains the largest part of the variance of the dependent variable is the first to enter the model.

To examine the robustness of the results, we divided the sample into three subgroups according to CAMBIOS scores and carried out a one-way ANOVA to study differences in HRV indices and SampEn-FC. In all analyses involving repeated measures, the Greenhouse–Geisser epsilon correction was applied to control for violation of the sphericity assumption. *Post hoc* pairwise mean comparisons were performed by using Bonferroni correction with a level of significance set at 0.05. All analyses were performed with SPSS v.15.0 (SPSS, Inc., Chicago, IL, United States).

## Results

### Correlations and Partial Correlations Between the CAMBIOS Test, HRV and SampEn-FCm

**FIGURE 1:**
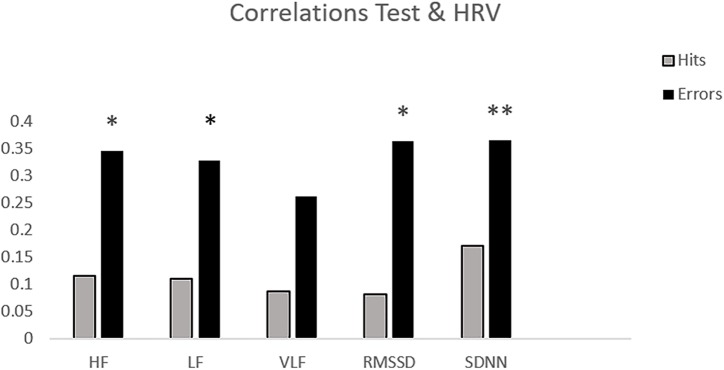
Bar chart of the correlations between HRV measures and subscores on CAMBIOS (corrects and errors). ^∗^*p* < 0.05; ^∗∗^*p* < 0.01 (two-tailed).

Table 1 shows the mean and standard deviation in CAMBIOS scores and HRV indices. The correlations between CAMBIOS and HRV indices revealed significant positive correlations between HF, LF, RMSSD, SDNN and the CAMBIOS Error subscore. No significant correlations between the CAMBIOS Hits subscore and HRV indices were found (Figure 1).

**Table 1:** Means and standard deviations (SD) of CAMBIOS and HRV indices.

	Hits	Errors	HF	LF	VLF	RMSSD	SDNN
Mean	0.597	0.123	218.524	288.576	185.669	45.314	59.456
*SD*	0.205	0.095	206.622	256.571	164.945	22.212	18.911


**FIGURE 2:**
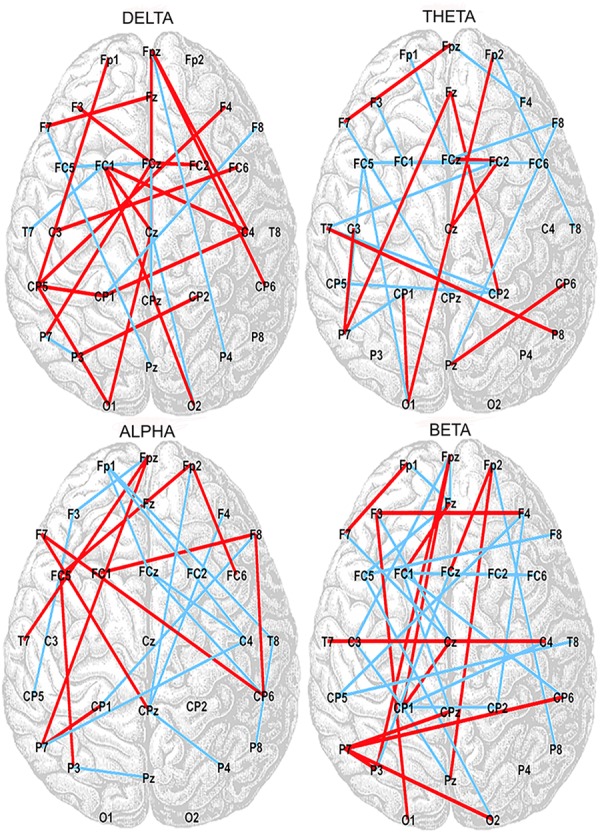
Positive (red lines) and negative (blue lines) correlations between SampEn-FC and Error subscore on the CAMBIOS test for delta, theta, alpha, and beta frequency bands.

Correlation analysis between the SampEn-FC of pairs of electrodes and the Error subscore of the CAMBIOS test revealed two networks for each frequency band (delta, theta, alpha, and beta). The positive network was composed of links that were positively correlated with the Error subscore, and the negative network was composed of links that were negatively correlated with the Error subscore (Figure 2). The beta band showed more links correlated with the Error subscore (negative correlations 21/positive correlations 35), followed by theta (15/24), alpha (14/24), and delta (7/25) (Table 2). Because the aim of this research was to study the relation between HRV, SampEn-FC and performance, the Hits subscore correlation was not obtained because HRV indices were not correlated in the previous analysis.

**Table 2:** Correlations between the SampEn-FC of pairs of electrodes and the Error subscore.

Delta		Theta		Alpha		Beta	
			
Links	Rho	Links	Rho	Links	Rho	Links	Rho
Fp1-Cp5	0.348	Fp1-FCz	-0.365	Fp1-C4	-0.435	Fp1-F7	0.367
F7-Fz	0.351	Fp2-T8	-0.355	Fp1-FCz	-0.545	Fp1-Fz	-0.440
F7-Pz	-0.341	Fp2-O1	0.414	Fp1-T8	-0.455	Fp2-FCz	0.344
F3-FCz	0.438	F7-CPz	-0.358	Fp2-Fc5	0.415	Fp2-Pz	0.418
F4-Cp5	0.387	F7-FPz	0.452	Fp2-Fc6	0.403	Fp2-P8	-0.345
F8-Cp5	-0.352	F3-Fc1	-0.368	Fp2-CPz	-0.347	F7-Cz	-0.417
Fc5-FCz	-0.369	Fz-Cp2	0.362	F7-CPz	0.345	F3-F4	0.401
Fc1-Cz	0.478	Fz-P7	0.412	F7-Cp6	0.486	F3-Cz	-0.384
Fc1-C4	0.388	F4-FPz	-0.392	F3-Cp5	-0.385	F3-O1	0.359
Fc1-O2	0.349	F8-FCz	-0.455	F3-FPz	-0.523	Fz-Fc5	-0.496
Fc2-FCz	0.621	F8-Pz	-0.549	F4-CPz	-0.371	Fz-Fc1	0.342
Fc6-C3	0.451	Fc5-Fc2	-0.356	F8-Fc1	0.444	Fz-FPz	-0.386
Cz-O1	0.498	Fc5-Fc6	-0.373	F8-Cp1	-0.384	F4-Cp5	-0.409
Cz-FPz	0.435	Fc5-P7	-0.403	F8-Cp6	0.353	F4-Cp2	-0.435
Cz-O2	-0.349	Fc5-O1	-0.426	Fc5-P3	0.421	F8-Fc5	-0.393
C4-Cp1	0.370	Fc2-T7	-0.417	T7-FPz	0.381	Fc5-O2	-0.345
C4-FPz	0.431	Fc2-Cz	0.423	C4-FCz	-0.368	Fc1-Fc6	-0.400
FCz-CPz	-0.463	Fc2-FCz	0.358	C4-P7	-0.372	Fc1-CPz	-0.456
FCz-P7	0.355	T7-Cp2	-0.366	FCz-Cp6	-0.459	Fc1-Cp6	-0.421
Cp5-Cp1	0.403	T7-P8	0.504	T8-P8	-0.420	T7-Cz	0.375
Cp5-O1	0.386	Cp5-Cp2	-0.397	Cp1-P7	0.342	C3-Cz	-0.389
Cp2-P3	0.423	Cp1-P7	-0.421	CPz-P4	-0.343	C3-C4	0.469
Cp6-FPz	0.348	Cp1-O1	0.408	P7-FPz	0.426	C3-Cp1	-0.396
P7-P3	-0.375	Cp6-Pz	0.416	P3-Pz	-0.379	C3-FPZ	-0.376
P4-FPz	-0.415					C4-Cp1	-0.386
						FCz-P3	-0.342
						T8-Cp5	-0.373
						Cp1-Cp2	-0.448
						Cp1-Pz	-0.355
						Cp1-FPz	0.345
						CPz-P7	0.367
						Cp6-P7	0.381
						P7-P3	0.350
						P7-O2	0.382
						P3-FPz	0.374


Because previous studies indicated that executive functions are positively correlated with frontal connectivity (Fox et al., 2006; Seeley et al., 2007), SampEn-FC means (SampEn-FCm) were calculated by averaging the electrodes for the positive and negative networks obtained for each frequency band. Six regions of interest were obtained by grouping the following electrodes: frontal (Fp1, FPz, Fp2, F7, F3, Fz, F4, and F8), central (Fc5, Fc1, FCz, Fc2, Fc6, C3, Cz, and C4), temporal left (T7), temporal right (T8), parietal (Cp5, Cp1, CPz, Cp2, Cp6, P7, P3, Pz, P4, and P8), and occipital (O1 and O2). One-way ANOVA with repeated measures was used to compare differences in SampEn-FCm in each region in the positive and negative networks in each frequency band. No significant differences in functional connectivity variability were found between different regions in the positive [delta *F*(3, 99) = 0.26, *p* > 0.05; $\eta_{p}^{2}$ = 0.01; theta *F*(4, 132) = 0.685, *p* > 0.05; $\eta_{p}^{2}$ = 0.02; alpha *F*(3, 99) = 0.506, *p* > 0.05; $\eta_{p}^{2}$ = 0.02; beta *F*(4, 132) = 0.821, *p* > 0.05; $\eta_{p}^{2}$ = 0.02] or negative network [delta *F*(4, 132) = 1.67, *p* > 0.05; $\eta_{p}^{2}$ = 0.05; theta *F*(5, 165) = 1.74, *p* > 0.05; $\eta_{p}^{2}$ = 0.05; alpha *F*(3, 99) = 0.07, *p* > 0.05; $\eta_{p}^{2}$ = 0.002; beta *F*(4, 132) = 0.06, *p* > 0.05; $\eta_{p}^{2}$ = 0.003].

Next, the SampEn-FCm of the whole brain was calculated from the positive and negative networks in each frequency band. In the positive network, the SampEn-FCm values were 2.531 in delta, 2.549 in theta, 2.538 in alpha, and 2.549 in beta. In the negative network, the SampEn-FCm values were 2.526 in delta, 2.578 in theta, 2.553 in alpha, and 2.523 in beta.

**FIGURE 3:**
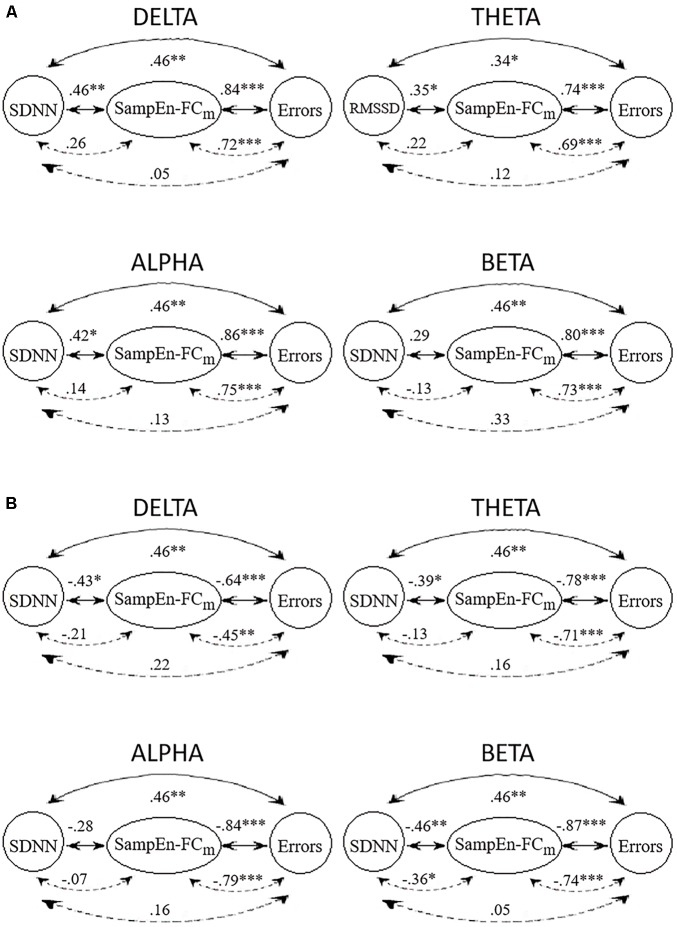
Correlations (full lines) and partial correlations (broken lines) among HRV index with higher significant correlations with the SampEn-FCm of positive networks **(A)** and negative networks **(B)** and Error subscore in delta, theta, alpha, and beta frequency bands. ^∗^*p* < 0.05; ^∗∗^*p* < 0.01; ^∗∗∗^*p* < 0.001 (two-tailed).

Figure 3 displays the Spearman correlations and partial correlations between HRV, SampEn-FCm and the Error subscore in all frequency bands. The SampEn-FCm values in the delta band and alpha band showed significant correlations with LF, SDNN and the Error subscore in the positive network (Figure 3A). The SampEn-FCm of the theta band correlated with RMSSD, while the Error subscore and SampEn-FCm of the beta band correlated with the Error subscore. In the negative network (Figure 3B), the SampEn-FCm values of the delta band and theta band correlated with SDNN and the Error subscore, that of the beta band correlated with HF, LF, SDNN and the Error subscore, and that of the alpha band correlated only with the Error subscore.

All frequency bands showed significant positive and negative correlations between SampEn-FCm and the Error subscore when HRV indices were partialled out. In contrast to results obtained for other frequency bands, partial correlations showed significant negative correlations between SampEn-FCm and SDNN when the CAMBIOS test was partialled out in the beta band.

In general, HRV indices and Error subscore correlations were weakened when the SampEn-FCm was partialled out. Similarly, HRV indices and SampEn-FCm correlations were weakened when the Error subscore was partialled out. However, SampEn-FCm and the Error subscore correlation did not decline when HRV indices were partialled out.

### Multiple Linear Regressions

Table 3 displays the standardized beta weights resulting from the stepwise regression analyses. The positive network in the delta and alpha bands and the HRV indices used as predictors revealed a single model, in which SampEn-FCm directly predicted the number of errors in the CAMBIOS test. Moreover, the positive network in the theta and beta bands and the HRV indices used as predictors showed that SampEn-FCm and LF directly predicted the Errors subscore. The negative network in the theta, alpha and beta and the HRV indices used as predictors revealed a single model, in which SampEn-FCm inversely predicted the Errors subscore. Furthermore, the negative network in the delta band and the HRV indices used as predictors showed that SampEn-FCm inversely predicted the Errors subscore, while RMSSD directly predicted the Errors subscore.

**Table 3:** Regression analyses for the prediction of the Error subscore from the physiological parameters assessed during rest (standardized beta weights).

Positive network	Error subscore	Negative network	Error subscore
**Delta**		**Delta**	
SampEn-FCm	0.764^***^	SampEn-FCm	-0.518^**^
HF	0.095	HF	0.289
LF	0.017	LF	0.173
RMSSD	0.137	RMSSD	0.322^*^
SDNN	0.044	SDNN	0.217
**Theta**		**Theta**	
SampEn-FCm	0.742^***^	SampEn-FCm	-0.742^***^
HF	0.131	HF	0.177
LF	0.258^*^	LF	0.161
RMSSD	0.091	RMSSD	0.154
SDNN	0.171	SDNN	0.140
**Alpha**		**Alpha**	
SampEn-FCm	0.787^***^	SampEn-FCm	-0.817^***^
HF	0.168	HF	0.145
LF	0.069	LF	0.077
RMSSD	0.164	RMSSD	0.183
SDNN	0.082	SDNN	0.121
**Beta**		**Beta**	
SampEn-FCm	0.733^***^	SampEn-FCm	-0.779^***^
HF	0.207	HF	0.058
LF	0.249^*^	LF	0.035
RMSSD	0.215	RMSSD	0.117
SDNN	0.226	SDNN	0.001


^∗^*p* < 0.05; ^∗∗^*p* < 0.01; ^∗∗∗^*p* < 0.001 (two-tailed).

### Inter-Group Differences

To test differences in RSVFC networks and HRV between participants with high, medium and low performance in the switching task, we divided the sample into three subgroups according to the distribution of the sample in terms of CAMBIOS Error scores (low-, medium-, and high-error groups).

**FIGURE 4:**
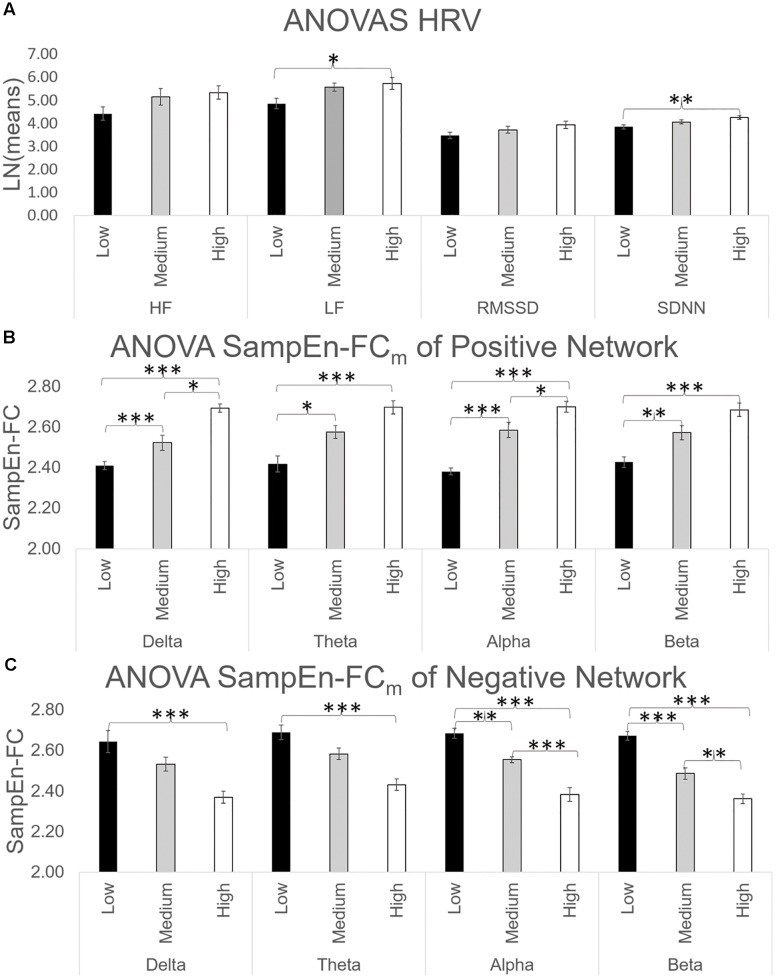
**(A)** ANOVA of HF, LF, RMSSD, and SDNN indices. The HRV indices were log-transformed to equalize the scales. **(B)** ANOVA of SampEn-FCm of positive networks obtained in Figure 2 for all frequency bands. **(C)** ANOVA of SampEn-FCm of negative networks obtained in Figure 2 for all frequency bands. ^∗^*p* < 0.05; ^∗∗^*p* < 0.01; ^∗∗∗^*p* < 0.001 (two-tailed).

The ANOVA results for HRV indices revealed significant effects of group on LF [*F*(2, 31) = 4.35, *p* < 0.05; $\eta_{p}^{2}$ = 0.219] and SDNN [*F*(2, 31) = 5.43, *p* < 0.01; $\eta_{p}^{2}$ = 0.260]. Bonferroni tests comparing the CAMBIOS Error scores between the three groups showed significant differences between the low and high-error groups in both HRV indices (all *p* < 0.05). No significant differences were found between the medium-error group and the other two groups. Figure 4A depicts the HF, LF, RMSSD, and SDNN indices corresponding to each group of CAMBIOS Error score stimuli. As shown, the high-error group showed more LF and SDNN power than did the low-error group.

ANOVAs for SampEn-FC means across four frequency bands in the positive network yielded significant differences in delta [*F*(2, 31) = 35.27, *p* < 0.001; $\eta_{p}^{2}$ = 0.695], theta [*F*(2, 31) = 15.81, *p* < 0.001; $\eta_{p}^{2}$ = 0.505], alpha [*F*(2, 31) = 43.87, *p* < 0.001; $\eta_{p}^{2}$ = 0.739] and beta [*F*(2, 31) = 19.29, *p* < 0.001; $\eta_{p}^{2}$ = 0.554]. As shown in Figure 4B, the Bonferroni test revealed that the high-error group had a higher SampEn-FC mean than the low-error group (*p* < 0.001).

Finally, ANOVAs for SampEn-FC means across the frequency bands in the negative network yielded significant differences in delta [*F*(2, 31) = 9.83, *p* < 0.001; $\eta_{p}^{2}$ = 0.388], theta [*F*(2, 31) = 15.95, *p* < 0.001; $\eta_{p}^{2}$ = 0.507], alpha [*F*(2, 31) = 33.18, *p* < 0.001; $\eta_{p}^{2}$ = 0.682] and beta [*F*(2, 31) = 46.74, *p* < 0.001; $\eta_{p}^{2}$ = 0.751]. The *post hoc* comparations yielded significant differences between the high-error group and low-error group in terms of the SampEn-FC mean (*p* < 0.001; Figure 4C) and between the medium-error group and the low-error group (*p* < 0.05).

## Discussion

The aim of the present study was to determine whether individual functional connectivity variability in the resting state (RSVFC) and HRV predicted performance on a cognitive task. Our results confirm that HRV significantly correlated with performance on tasks that involved cognitive flexibility. Moreover, RSVFC correlated with performance on the tasks in all frequency bands. However, partial correlation showed that the correlation of heart rate fluctuations with cognitive flexibility was indirect and was mediated by functional connectivity variability, while the correlation between functional connectivity variability and task performance remained significant when the heart rate fluctuation index was partialled out. Finally, these results were confirmed when participants were divided into three subgroups according to their behavioral performance on the task. Significant differences were found between the high-performance and low-performance test subgroups on RSVFC, LF, and SDNN indices.

The CAMBIOS test assesses the ability to concentrate while attending to several changing conditions, e.g., the cognitive flexibility to determine if different changes are fulfilled and the speed of this process (Contreras et al., 2003). Our results revealed positive correlations between HRV (HF, LF, RMSSD, and SDNN) and the Error subscore of the CAMBIOS test and between CAMBIOS test performance and functional connectivity variability in the resting state. These results are inconsistent with the classic assumption of an association between higher levels of cardiac vagal tone and cognitive performance (Albinet et al., 2010, 2016; Stenfors et al., 2016). Several studies found a positive correlation between higher levels of cardiac vagal tone and the number of errors on a cognitive task and between HRV indices and attentional function during both resting and task periods (Duschek et al., 2009; Hovland et al., 2012). Duschek et al. (2009) hypothesized that difficult tasks or tasks executed under time pressure demand higher energetic resources. Thus, the degree of central activation depends on the difficulty of the task, and the association between resting HRV and executive function depends on the requirements of the cognitive test. Therefore, the inverse association between resting HRV and CAMBIOS performance suggests that a pattern of cardiovascular adjustment, including enhanced sympathetic and reduced vagal cardiovascular influences, may induce an adaptive state associated with improved cognitive flexibility functioning.

The relationship between functional connectivity variability in the resting state and CAMBIOS test performance revealed two distinct networks; one network comprises regions that presented positive correlations between variability and the task Error subscore, while other regions exhibited negative correlations. No significant differences were found in functional connectivity variability between the electrode groups of interest (frontal, central, temporal left, temporal right, parietal, and occipital) in either network in each frequency band. Previous studies indicated more dynamic connectivity of the fronto-parietal localization in response to different frequency ranges during cognitive task performance, and this connectivity is related to better cognitive performance (González-Hernández et al., 2002; Fornito et al., 2012; Cole et al., 2013; Monti et al., 2014; Beatty et al., 2015; Vatansever et al., 2015). However, research on the dynamic connectivity between the fronto-parietal localization in the resting state with respect to the prediction of cognitive functioning yields mixed results. Both positive and negative correlations between resting-state dynamic connectivity and cognitive performance have been reported (Jia et al., 2014; Kucyi and Davis, 2014; Sadaghiani et al., 2015). Our results yielded no differences in variability between the electrode groups of interest and rhythms in resting states in relation to the performance task. Although this result could be due to the low spatial resolution of EEG, multiple brain regions are activated in parallel, and these regions do not generally display pure oscillations in resting conditions (Mantini et al., 2007; de Pasquale et al., 2010; Tagliazucchi et al., 2012). In fact, spontaneous brain activity in the resting state is organized in a finite set of spatiotemporal patterns that are linked to EEG brain rhythms (Mantini et al., 2007). Conversely, a combination of delta, theta, alpha, beta, and gamma rhythms are ascribed to brain networks during the resting state. This information leads us to think that high variability between cortical localization and rhythms could facilitate flexibility in adapting processing to changing tasks. The variability of functional connectivity is higher in healthy adults (showing better performance in cognitive tasks) than in older adults who show worse cognitive performance (McIntosh et al., 2008, 2010; Garrett et al., 2011), and default mode network connectivity (a network activated in the resting state Laufs et al., 2003; Raichle and Snyder, 2007) makes the greatest contribution to executive function/cognitive flexibility prediction (Liu et al., 2018). Our results suggest that the levels of variability in connectivity between localization are related to the effectiveness of performance and suggest beneficial effects of higher brain signal variability in general.

Very compelling evidence suggests that HRV is an indicator of the adaptation of the ANS to a variety of psychological and behavioral situations (Thayer et al., 2010; Zahn et al., 2016). Higher HRV is associated with performance on several cognitive tasks involving attention, working memory, and inhibitory control (Thayer et al., 2005; Duschek et al., 2009; Hovland et al., 2012). Similarly, direct correlations show that HRV or functional connectivity networks in the resting state are related to cognitive performance (Hansen et al., 2003; Saus et al., 2006; Chang et al., 2013b; Thompson et al., 2016). Our results revealed that HRV and RSVFC were related to the CAMBIOS performance, however, partial correlations revealed that RSVFC in all frequency ranges mediated the relationship between HRV and the cognitive test. Regression analyses indicated that RSVFC accounted for the largest portion of CAMBIOS performance variance in all frequencies. Although direct correlations among cognitive flexibility, neuronal and heartbeat variability are significant, partial correlations and multiple linear regressions suggest that the relation between heartbeat and cognitive performance is mediated by neuronal oscillations. Palva et al. (2013) found similar results using power-law form, long-range, temporal correlations in the resting state and during stimulus detection tasks. Moreover, neuronal networks that correlated with performance in the resting state were similar to networks during the task. Our results extend these findings to cognitive performance, suggesting that the levels of brain signal variability might predict cognitive flexibility in a cognitive task.

ANOVA confirmed that HRV and functional connectivity variability are associated with cognitive performance. The results yielded significant differences among the high-, medium- and low-Error subscore groups in LF and SDNN indices and RSVFC (positive and negative networks). Thus, participants with more errors on the CAMBIOS test showed more LF and SDNN, more variability in the positive network and less variability in the negative network in all frequency ranges. Other studies have compared resting HRV (Hansen et al., 2004; Albinet et al., 2010, 2016) and the variability of functional connectivity (Takeuchi et al., 2011; Martínez et al., 2013; Thompson et al., 2016) between groups with high and low cognitive performance using ANOVA. Higher resting HRV predicts good performance in cognitive tasks (Albinet et al., 2016; Stenfors et al., 2016). However, some studies suggest that the classic assumption of an association between higher resting levels of cardiac vagal tone and improved cognitive performance does not universally hold true. Thus, Hovland et al. (2012) found that HF-HRV in the resting state was positively correlated with the total errors and perseverative errors in the Wisconsin Card Sorting Test and with inhibition errors in the Color-Word Interference Test. Other studies have used batteries of cognitive tests, and they observed that not all tests of the battery correlated with HRV and the resting state (Jennings et al., 2015; Stenfors et al., 2016). The CAMBIOS test is a task with a high cognitive load that assesses the ability to handle a changing condition, similar to the Wisconsin Card Sorting Test, Color-Word Interference Test and Test D2. Duschek et al. (2009) hypothesized that the association between resting HRV and executive function performance may depend on the difficulty of the cognitive test when the brain is challenged. Therefore, more demanding tasks involve more cerebral resources, eliciting more heart rate activity.

In the future, replicating the current study using other cognitive tasks in which resting HRV has positive correlations with the studied hits would be interesting. For example, a continuous performance test or working memory test could be used because in these tasks, a high HRV group showed a faster mean reaction time, more correct responses and fewer errors than a low HRV group (Hansen et al., 2003, 2004). Partial correlations between HRV and these tasks when neuronal oscillations are partialled out have not been studied. The current study could be replicated using different cognitive tasks if interconnections between the CNS and ANS are influenced by the type of task.

In this study, the analysis of EEG sources was not possible because 32 electrodes were insufficient for the accurate localization of EEG sources (Lantz et al., 2003). Future studies should include EEG systems with more electrodes and source localization analysis. Source localization methods allow quantitative prediction of the locations of EEG activity inside the brain. Thus, EEG source localization analysis is necessary for determining brain areas related to cognitive performance and heart rate oscillations with enhanced accuracy.

The neurovisceral model proposes that the relationship between the CNS and ANS is reciprocally interconnected such that information flows bidirectionally (Thayer and Lane, 2009; Smith et al., 2017). Our results seem to show that HRV, functional connectivity variability and cognitive flexibility are related between the CNS and ANS. Nevertheless, the relationship between HRV and cognitive performance is determined by the type of task and mediated by the functional connectivity variability of the brain. More studies using different cognitive tasks and paradigms are necessary to establish causal relations between these variables.

## Author Contributions

GA, JV, BR, PM, and MM contributed significantly to the design of the study. GA performed the data collection. GA and MM performed the data analysis and wrote most of the manuscript. and JV, BR, and PM critically revised important parts of the manuscript.

## Conflict of Interest Statement

The authors declare that the research was conducted in the absence of any commercial or financial relationships that could be construed as a potential conflict of interest.
